# The Problem of Wound Healing in Diabetes—From Molecular Pathways to the Design of an Animal Model

**DOI:** 10.3390/ijms23147930

**Published:** 2022-07-19

**Authors:** Mateusz Mieczkowski, Beata Mrozikiewicz-Rakowska, Michał Kowara, Marcin Kleibert, Leszek Czupryniak

**Affiliations:** 1 Department of Diabetology and Internal Diseases, Medical University of Warsaw, 02-097 Warsaw, Poland; mateusz.mieczkowski@gmail.com (M.M.); marcin.kleibert@gmail.com (M.K.); leszek.czupryniak@wum.edu.pl (L.C.); 2 Chair and Department of Experimental and Clinical Physiology, Laboratory of Centre for Preclinical Research, Medical University of Warsaw, Banacha 1b, 02-097 Warsaw, Poland; michal.kowara@wum.edu.pl

**Keywords:** animal model, molecular mechanism, diabetic foot syndrome, chronic wound

## Abstract

Chronic wounds are becoming an increasingly common clinical problem due to an aging population and an increased incidence of diabetes, atherosclerosis, and venous insufficiency, which are the conditions that impair and delay the healing process. Patients with diabetes constitute a group of subjects in whom the healing process is particularly prolonged regardless of its initial etiology. Circulatory dysfunction, both at the microvascular and macrovascular levels, is a leading factor in delaying or precluding wound healing in diabetes. The prolonged period of wound healing increases the risk of complications such as the development of infection, including sepsis and even amputation. Currently, many substances applied topically or systemically are supposed to accelerate the process of wound regeneration and finally wound closure. The role of clinical trials and preclinical studies, including research based on animal models, is to create safe medicinal products and ensure the fastest possible healing. To achieve this goal and minimize the wide-ranging burdens associated with conducting clinical trials, a correct animal model is needed to replicate the wound conditions in patients with diabetes as closely as possible. The aim of the paper is to summarize the most important molecular pathways which are impaired in the hyperglycemic state in the context of designing an animal model of diabetic chronic wounds. The authors focus on research optimization, including economic aspects and model reproducibility, as well as the ethical dimension of minimizing the suffering of research subjects according to the 3 Rs principle (Replacement, Reduction, Refinement).

## 1. Introduction

Epidemiological data clearly show that diabetes mellitus (DM) has long been a challenge for the representatives of the medical world and healthcare-financing entities. Estimates show that the number of DM cases and diabetic complications worldwide is increasing, including difficult-to-heal wounds, with the most representative example being diabetic foot syndrome (DFS). It is assessed that during the lifetime of a patient with DM, the probability of DFS occurrence ranges from 5 to 25%, according to various authors. DFS is found in 10 to 20% of patients with diabetes, and detailed data from the USA report even 25% [1,2]. There are no exact data on what percentage of patients with DM have wounds of ischemic etiology or those resulting from venous insufficiency. The lack of such precise epidemiologic data may be due to the fact that the abovementioned etiologic factors of wounds, i.e., atherosclerotic lesions or venous insufficiency, which is less frequent, may coexist in a patient with DM at different rates simultaneously. In addition, impaired wound healing in diabetic patients results from comorbidities. Cellulitis is often the triggering factor for wound formation, which might be an indicator of increasing swelling resulting from heart or kidney failure, a large percentage of which is due to changes caused by DM. Additionally, patients with DM are more vulnerable to injuries due to neuropathy. Regardless of its etiology, any wound that develops initially on the lower leg may become a precursor to DFS when it spreads below the ankle. When ulceration becomes infected, especially in the case of DFS due to impaired immune response in these patients, it can generate complications such as sepsis, amputation, and even death. In patients with DM who developed active ulceration within five years, the mortality rate has been reported to be as high as 40% [3]. Considering mortality following amputation, the figures are even more terrifying since they range from 13% to 40% at one year, 35% to 65% at three years, and 39% to even 80% at five years [1]. For this reason, research is conducted in scientific laboratories worldwide to produce molecules of both local and systemic action, which could accelerate wound healing and inhibit the development of inflammation at the lowest possible stage. Before these molecules can be used in humans, it is necessary to carry out studies in an animal model, which could demonstrate the safety and efficacy of such molecules. To achieve this goal, the protocol of animal studies should be optimized. Moreover, the variety of the choice of models for preclinical studies and related problems closely reflecting the pathophysiology of wound development in human DM can be challenging. In this paper, we summarize the current information related to the pathogenesis of impaired wound healing in DM in the context of the design of an animal model. Additionally, the paper presents the most important practical aspects of designing and selecting an animal model for preclinical studies on wound healing in DM. 

## 2. Molecular Pathways of the Development of Chronic Wounds in DM

**Figure 1:**
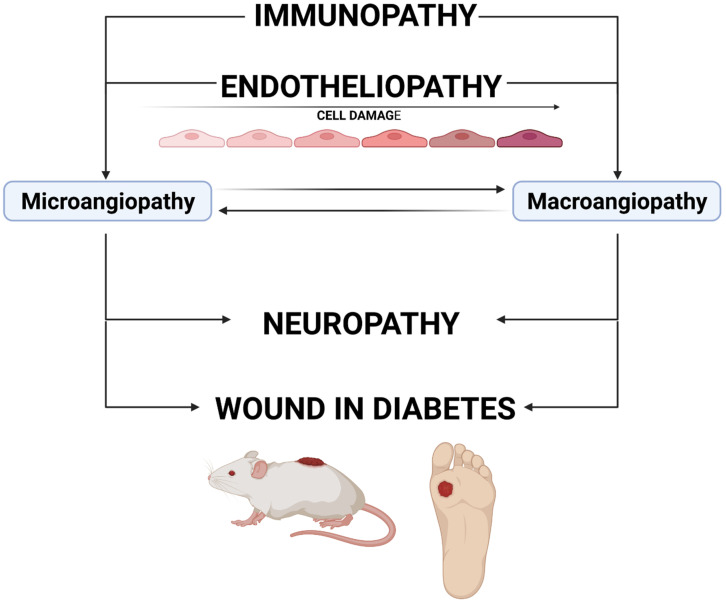
The mechanism of wound development in diabetes. Immunopathy and endotheliopathy are the causes of the development of microangiopathy and macroangiopathy. The impairment of blood supply (mainly lesions in the vasa nervorum) can cause neuropathy. All these pathologies lead to an increased risk of wound development and a delayed healing process. Created with BioRender.com.

The source of impaired wound healing in DM is related to several overlapping mechanisms, presented in Figure 1. Endotheliopathy, which results in micro- and macrovascular changes, can lead to neuropathic changes, which significantly increase the risk of injury and delay in wound healing [4]. Macroangiopathic lesions, which usually occur later than microvascular changes, affect the supply of nutrients to the wound during vascular complications in DM. Importantly, although the studies investigating microvascular complications of DM, such as diabetic retinopathy, diabetic nephropathy and neuropathy, have used various animal models (e.g., streptozotocin or alloxan-induced DM in mice and rats, DM induced by transgenic procedures as in Akita mice), the mechanism leading to microvascular complications (microangiopathy) and created in these models is the same—hyperglycemia and other important changes such as impaired immune response or altered intracellular pathways [5]. Investigations of macrovascular changes are focused on animal models of specific macrovascular lesions, e.g., myocardial infarction or stroke. At a certain stage of the development of microangiopathic and macroangiopathic wound progression, revascularization is the only chance for wound healing. As yet, no vascular surgery techniques have allowed the restoration of the flow in microcirculation vessels (known as resistance subcutaneous arteries) due to the mostly irreversible nature of vessel wall remodeling and thrombotic and embolic closure. Firstly, the aim of the current studies should be to find optimal and possibly non-invasive assessment methods for the flow in the microvascular bed. This is extremely important when the changes in small arteries are potentially reversible, before significant thickening of the basement membrane and thrombotic and embolic occlusions occur. New MRI-based techniques used in preclinical studies on cerebral microcirculation in rodents might also be useful in diabetic wound studies [6]. Secondly, searching for microvascular revascularization methods and/or molecules and drugs preventing the occurrence of thrombotic and embolic changes and basal membrane thickening should be independent research directions [7,8]. Studies on both of these problems require designing an adequate animal model of wounds in DM, which will imitate impairment related to DM.

### 2.1. Diabetic Endotheliopathy

**Figure 2:**
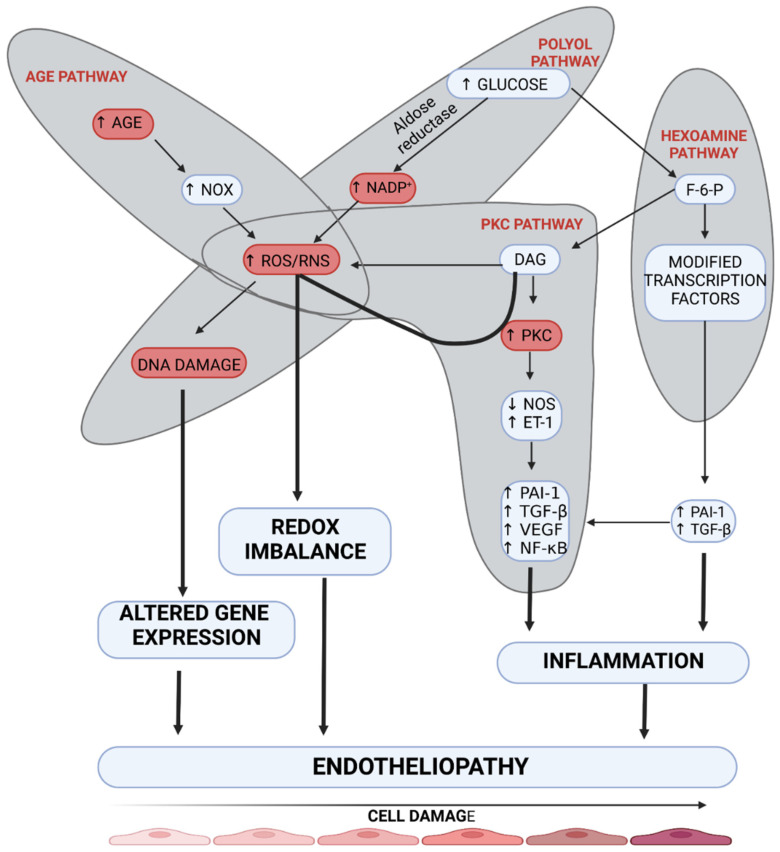
The mechanism of the development of endotheliopathy. Four main pathways are involved in the damage to endothelial cells (AGE pathway, polyol pathway, PKC pathway, and hexosamine pathway). Overactivation of these molecular pathways leads to inflammation, redox imbalance, and altered gene expression, which results in endotheliopathy. AGE—advanced glycation end-product, DAG—diacylglycerol, DNA—deoxyribonucleic acid, ET-1—endothelin-1, F-6-P—fructose 6-phosphate, NADP+—nicotinamide adenine dinucleotide phosphate, NF-κB—nuclear factor kappa-light-chain-enhancer of activated B cells, NOS—nitric oxide synthase, NOx—nitrogen oxides, PAI-1—plasminogen activator inhibitor-1, PKC—protein kinase C, RNS—reactive nitrogen species, ROS—reactive oxygen species, TGF-β—transforming growth factor β, VEGF—vascular-endothelial growth factor. Created with BioRender.com.

Endotheliopathy plays a leading role due to impaired tissue metabolism in DM [9]. It is related to a disturbed reactivity of the vessel wall, which in long-term DM causes not only an impaired flow to distal parts but also, paradoxically, an overflow at the arteriolar level within the tissue, due to the formation of arteriovenous anastomoses (AVAs) [10,11]. Hyperglycemia affects endotheliopathy through pathways leading to microangiopathy, such as polyol, hexosamine, advanced glycation end-products (AGEs), and protein kinase C (PKC) pathways (Figure 2) [12]. Of these four pathways, the PKC pathway seems to affect endothelial vascular dysregulation most directly. Hyperglycemia may increase the synthesis of regulatory enzymes belonging to the PKC family independently or through increased diacylglycerol (DAG) synthesis. Activation of these enzymes results in endothelium-dependent dysfunction of microarterioles regulating contractility of the smooth muscle of vessels supplying distal tissue areas [13]. Moreover, endothelial dysfunction of small vessels results from the inhibition of the nitric oxide (NO) and endothelium-derived hyperpolarizing factor (EDHF) pathways and enhanced production of endothelin-1 and oxygen-free radicals [14]. Khamasi et al. showed that patients with DFS were characterized by increased levels of PKCδ, which was associated with inhibition of insulin signaling. This can be associated with delayed wound healing due to a restriction of oxygen and nutrient supply by impaired arteriole relaxation [15]. The abovementioned changes are also observed in animal models in studies on DM. A study on a mouse model showed that hyperglycemia (developed in streptozotocin-induced diabetic mice) caused a significant increase in PKC-δ expression, which resulted in subsequent SHP-1 activation, PDGF downstream signaling reduction, cellular apoptosis, and the presence of acellular capillaries within the retina compared to non-diabetic control mice. These changes in diabetic mice were not reversible by insulin treatment. However, the aforementioned changes were not developed in transgenic Prkcd −/− mice that lacked PKC-δ signaling. This study highlights the role of PKC-δ in the development of endotheliopathy [16]. In addition, a study on rat mesangial cells demonstrated that advanced glycation end products (AGEs) induced intracellular oxidation stress, which contributes to the development of pathological changes in DM [17]. Moreover, hyperglycemia in streptozotocin-induced diabetic mice causes neuronal injury, especially in the frontal cortex and amygdala, through inducible nitric oxide synthase (iNOS) overexpression [18]. The imitation of this process can be achieved by induction of hyperglycemia, which can induce enzyme synthesis and overactivation of the PKC pathway. Importantly, the PKC pathway, which seems to be crucial in the pathogenesis of DM is also up-regulated in ob/ob mice, which reflects type 2 DM, not only in streptozotocin-induced diabetic mice [18].

The polyol pathway leads to endotheliopathy through the decreased supply of NO in dysfunctional vessels by reducing NADPH in the cytosol. NADPH is necessary for the regeneration of glutathione, which has antioxidant properties, and also for the production of nitric oxide synthase (NOS) [19]. In the case of cytosolic deficiency of NADPH, uncoupling of eNOS does not occur, which results in decreased production of NO and increased production of oxygen free radicals, including ONOO^−^ and O^2−^. The formation of ROS products exacerbates endothelial cell dysfunction, thus mediating DNA damage [20]. Increased activity of the hexosamine pathway occurs due to hyperglycemia. The final stage of this pathway is related to the formation of UDP-N-Acetylglucosamine, which results in increased expression of TGF-β1 and PAI-1. Under hyperglycemic conditions, a four-fold increase is reported in O-GlcNAcylation of the transcription factor Sp1, which participates in the development of endotheliopathy by activating TGF-β1 and PAI-1 in arterial endothelial cells and the PAI-1 promoter in vascular smooth muscle cells. Additionally, activation of receptors for AGEs (RAGE) triggers NADPH oxidase, which enhances redox reactions and consequently causes further endothelial damage [21]. Not only does AGE cause damage to endothelial cells, but it also causes changes in the structure and function of extracellular (ECM) and intracellular (ICM) matrix proteins [19]. These processes delay wound healing due to the impaired activation of fibroblasts which secrete the ECM proteins. Moreover, it should be stressed that RAGE and PKC pathway upregulation results in increased NF-κB factor signaling, mediating the synthesis of endothelin-1 and ICAM adhesion molecules, which results in increased migration of immune cells [22]. A study on obese hyperglycemic mice showed that administration of sEH inhibitor t-AUCB inhibited the degradation of epoxyeicosatrienoic acids (EETs) and could increase EDHF secretion, which resulted in the inhibition of microangiopathic lesion formation [23]. Animal models should imitate the impaired activation of the EDHF pathway due to its essential role in the pathogenesis of microangiopathy.

### 2.2. Diabetic Neuropathy

Most of the processes responsible for the development of endotheliopathy, including increased polyol pathway activity, AGE formation, and the PKC pathway, simultaneously lead to the development of neuropathy [24]. In addition to hyperglycemia, other factors such as glycemic variability, lipid disturbances, smoking, and alcohol abuse may influence the occurrence and severity of neuropathy symptoms [25]. Damage to the vasa nervorum, which supply various nerve fibers, such as C-type and delta fibers responsible for specific functions of the nervous system, is mainly responsible for impaired wound healing in DM and its recurrence, especially in DFS [26]. This highlights the importance of imitating neuropathic changes in the design of the animal model [27]. Impaired microcirculation in the skin in patients with DM occurs due to damage to the vasa nervorum caused by thickening of the basal membrane of those arteries and their persistent occlusion by microclots. This leads to a decrease in oxygen partial pressure in patients with DFS despite preserved flow in the vascular bed [28]. This change results in sustained ischemia of distally located tissues and persistently decreased pH in this area, which activates Acid-Sensing Ion Channel-3 (ASIC3), responsible for transmitting pain signals [29,30]. However, its transmission may be impaired due to neuropathy. A study on nociception in Ins2+/Akita mice (a hereditary model of DM in which the disease develops in young animals after weaning) revealed reduced action potential discharge in mechanonociceptors compared with wild-type animals, which was reversed by restoration of normal glucose levels [31].

Besides autonomic responses, unmyelinated C-type fibers are responsible for pain perception. Up to 70% of multifunctional C-fibers are present in the skin [32,33]. Their stimulation initiates neurogenic inflammation in response to neuropeptides released from sensory nerve endings. These neuropeptides include substance P and calcitonin gene-related peptide (CGRP), and their role is to regulate the activation of dendritic cells, mast cells, and T cells. Microangiopathy, which causes impaired secretion of these neuropeptides and neurotransmitters, results in not receiving signals by the immune system, whose cells cannot undertake their functions [32,33,34]. Thus, the cell signaling processes responsible for a normal immune response are strongly disrupted [35]. In the natural course of DM, particularly if long-standing and poorly compensated, the C-fiber function permanently deteriorates, inevitably causing impaired neurogenic inflammation responsible for neuropathic pain and the inadequate response to inflammation [36]. In this process, especially TRPV1, which is the transient receptor potential vanilloid subfamily member, plays a significant role [29]. TRPV1 receptors are located in sensory nerve fibers and some vascular endothelial and smooth muscle cells. Of note, they participate in the integration of pain stimulus transmission. Besides inflammation in the wound, activation of TRPV1 is an additional factor that triggers pain sensation. Paradoxically, due to this neurogenic damage and due to lesions, the transmission of information about the emerging features of infection in a wound occurs with a delay or does not occur at all (e.g., in DFS). With regard to the immune changes occurring in the microcirculation in DM, the term immunocompromised district theory has been adopted [37].

### 2.3. Diabetic Immunopathy

**Figure 3:**
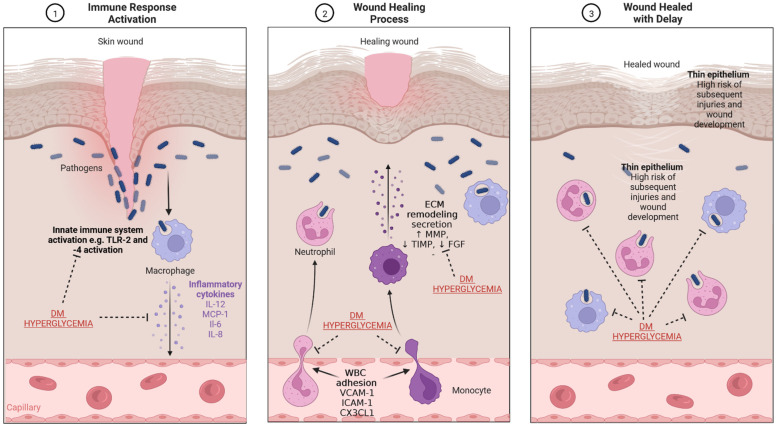
The mechanism of wound healing and impairment related to DM and hyperglycemia. (**1**) Immune response activation—the presence of pathogens, which enter through the damaged epithelium, leads to activation of innate immune response and secretion of inflammatory cytokines. (**2**) Wound healing process—secreted cytokines stimulate WBC diapedesis; macrophages and monocytes secrete substances which lead to re-epithelialization and ECM production and remodeling. (**3**) Wound healed with delay—the impaired physiological processes related to wound healing in diabetes result in the presence of thinner epithelium and an increased risk of infection. CX3CL1-chemokine (C-X3-C motif) ligand 1, DM—diabetes mellitus, FGF—fibroblast growth factor, ICAM-1—intercellular adhesion molecule 1, IL—interleukin, MCP-1—monocyte chemoattractant protein-1, MMP—matrix metalloproteinase, TIMP—tissue inhibitor of metalloproteinase, TLR-2—toll-like receptor 2, WBC—white blood cells, VCAM-1—vascular cell adhesion molecule-1. Created with BioRender.com.

Impaired wound healing in the course of ulceration results most often from an ongoing infectious process. A typical feature of chronic wounds is an increased concentration of metalloproteinases, which causes damage to the formation of granulation tissue [38]. A chronic wound in a hyperglycemic environment is subjected to special conditions. Due to the impaired functioning of immune system cells in this environment, some processes can be overactivated, with simultaneous underactivation of others. The immune system plays an important role in sustaining subclinical inflammation under conditions of developing hyperglycemia, as seen in individuals with obesity or in those who have already developed type 2 DM (Figure 3). It seems that hyperglycemia causes significant impairment of cell-dependent and humoral defense functions, which results in the long-term persistence of bacteria in the biofilm form in wounds in diabetic patients. Additionally, decreased myeloperoxidase and superoxide dismutase activities have been demonstrated in azurophilic granules of neutrophils in patients with persistent long-term hyperglycemia, which also causes delayed wound healing. Moreover, the aerobic mechanisms (production of ROS and respiratory burst) responsible for microbial inactivation by neutrophil granulocytes are impaired in DM. This is caused by, among others, excessive activation of the polyol pathway in DM, inducing NADPH deficiency, which prevents the generation of oxygen free radicals. It results in an impaired non-specific removal of pathogens, which is one of the key mechanisms of innate immunity. Furthermore, reduced activity of insulin-dependent enzymes in neutrophil granulocytes, causing impaired neutrophil migration, is also affected by relative insulin deficiency associated with insulin resistance in type 2 DM, or its complete absence in type 1 DM. Monocyte- and macrophage-dependent processes, such as chemotaxis and phagocytosis, are also impaired [39,40,41]. A decrease in adaptive immunity is observed due to the impaired activation of antigen-presenting cells (APCs). In the hyperglycemic milieu, increased mast cell degradation and increased recruitment of proinflammatory M1 macrophages were also shown to be responsible for the impaired cellular response and prolonged inflammation, which can also cause a delay in wound healing [42]. All the above processes are collectively defined as diabetic immunopathy [4]. It is important to imitate all these changes during the design of animal models due to their vital role in wound healing. Although diabetes also causes changes to the immune system in animals, these changes differ from the corresponding changes in humans. First, immune systems of mice or rats do not perfectly reflect the human immune system (e.g., there are two main subpopulations of monocytes in mice i.e., Ly6Chi and Ly6Clo) [43]. Second, it is difficult to extrapolate the exact pathogenesis of type 2 DM or even type 1 DM from humans to mice. For example, streptozotocin-induced DM, which is a common mice model, does not reflect autoimmune processes in the islets because it causes chemical (but not autoimmune) cell destruction. Therefore, developing new models and modifying the currently existing models in order to reflect immune processes in a more accurate way would be warranted. For instance, a model showing the experimental autoimmune reaction against pancreatic islets could reflect the natural pathogenesis of type 1 DM. Experience from similar models such as experimental autoimmune encephalomyelitis (EAE), a rodent model used in studies on multiple sclerosis, would be useful in generation of the above-mentioned hypothetical model [44].

## 3. Large Vessel Atherosclerotic Lesion in DM

The nature of atherosclerotic lesions in DM results not only from their much faster development compared to the general population but also from a subsequent predilection for lesions to be located in the intima and the media [45]. The diffuse character of changes in DM, which causes faster involvement of distal vessel segments than in patients with atherosclerosis without diabetes, is also noteworthy [46]. The reason for this difference is still not fully understood and needs further research. Therapeutic interventions by vascular surgeons or interventional radiologists do not always result in the expected revascularization outcomes due to the characteristic location of atherosclerotic lesions in DM.

### 3.1. Endotheliopathy as the Primary Factor Promoting Atherosclerosis

An impaired activation of many overlapping processes is the molecular basis of such a phenotype of changes as the ones observed in DM. Diabetic endotheliopathy is the initial process that facilitates the formation of vascular stenosis that initiates atherosclerotic plaque formation. Endothelial dysfunction is initiated not only by hyperglycemia but also by other factors such as increased arterial pressure, causing turbulent flow in vessels, high concentration of oxidized LDL, high concentrations of free oxygen radicals as well as homocysteine, genetic factors, inflammatory changes resulting from viral or bacterial infections, and the response to proinflammatory cytokines [47]. One of the final effects of these changes is a reduced NO synthesis in the endothelium, which leads to impaired reactivity of the vessel wall and a reduced antiatherosclerotic effect on plaque formation. The ability of optimal NO secretion depends on numerous factors such as the availability of tetrahydrobiopterin (BH4) and the activity of enzyme heme oxygenase-1 (HO-1). Their vital role in the homeostasis of NO was adopted for designing one of the mouse models of atherosclerosis development by knocking out the gene of the enzyme that participates in NO production (human HO-1 deficiency and Hmox1 −/− mice) [48]. This model seems to imitate the vascular changes but does not induce hyperglycemia. Therefore, it cannot be used as the model for the wound healing process in DM unless it is combined with other mutations or interventions which cause hyperglycemia. NO deficiency is not always the initiating factor for atherosclerotic lesions. Some studies have shown that NO synthesis may increase due to a higher concentration of free oxygen radicals, which was observed in patients with DM where abnormal vascular reactivity to NO was found [49]. Despite paradoxically increased NO production, endothelial cells showed simultaneously enhanced production of O^2−^ when treated with high concentrations of glucose [50]. This phenomenon requires further research to explain the dualistic role of glucose in these processes. Despite the above, the hyperglycemia milieu is a specific example of the potentially destructive effect of oxygen free radicals on the development of endotheliopathy [51]. The role of nitric oxide in the pathogenesis of DM has been widely investigated in animal models. An interesting study on neuronal nitric oxide synthase (nNOS) expression in pancreatic islets was performed on a rat model. It revealed that nNOS levels in beta cells disappeared after 12 h from the onset of DM [52]. 

The adhesion of platelets to damaged endothelial cells is one of the factors enabling the formation of atherosclerotic plaque and embolism. In DM, this process is enhanced by increased stimulation of growth factors, fibrin, thromboxane A2, integrins, and P-selectin. Activated platelets secrete numerous molecules which maintain local inflammation by stimulating endothelial cells. This leads to a constant mutual reaction between the endothelial cells and the atherosclerotic plaque, including the transformation of monocytes into macrophages by enhancing diapedesis [53]. 

### 3.2. Subclinical Inflammation as the Trigger of the Atherosclerotic Process

#### 3.2.1. Adhesion Molecules

Moreover, according to the current views, this subclinical inflammation is responsible for initiating and maintaining the atherosclerotic process. Indeed, there is a relationship between the activity of the innate and adaptive immune responses and the propagation of atherosclerotic lesions, which is particularly seen in DM. The innate immune response is activated by the subendothelial accumulation of cholesterol deposits and foam cells (fat-laden cells with an M2 macrophage-like phenotype), oxidation, and glycation [47,54,55]. It has been shown that the atherosclerotic plaque contains activated B cells, T cells, macrophages, mast cells, and dendritic cells that also send signals to the surrounding lymph nodes and around the area of the vascular lesion. The permanently damaged vessel wall becomes more permeable to leukocytes adhering to endothelial cells, mainly due to vascular (VCAM-1) and intercellular (ICAM-1) adhesion molecules [56]. The process of leukocyte adhesion and migration is also possibly due to chemokines such as fractalkine CX3CL1, which maintains the migration of macrophages to the site of the vessel wall and causes its damage [57]. Increased blood levels of VCAM and ICAM were observed in clinical conditions related to subclinical inflammation and hyperinsulinemia associated with insulin resistance, such as obesity or pre-DM [58,59]. It seems that peroxisome proliferator-activated receptors (PPARs), which are present in the walls of endothelial cells and monocytes, macrophages, and T cells, are one of the key regulators of this process. Oliver et al. showed that the use of PPAR-activating molecules (GW501516) in DM leads to the inhibition of the atherosclerotic process and the improvement of the lipid profile (increase in plasma HDL-C and decreased plasma triglyceride, LDL-C and insulin levels) [60,61]. PPAR pathways with KLF5 also participate in the development of diabetic cardiomyopathy, which has been demonstrated on transgenic mice [62].

#### 3.2.2. Toll-like Receptors 

In DM, chronic inflammation is responsible for the long-lasting wound healing process, which is another factor stimulating the development of atherosclerotic lesions. The activation of Toll-like receptors (TLRs) in the damaged tissue that are part of the innate immune system is the phenomenon linking these two processes [63]. During injury and infection, e.g., due to DFS, the TLRs are stimulated and mediate the expression of proinflammatory cytokines. In humans, increased expression of TLR receptors in the atherosclerotic plaque has been demonstrated [64]. In an ApoE −/− mouse model of atherosclerosis, deprivation of TLR4 receptors resulted in a reduction in atherosclerotic severity, with an associated decrease in the circulating levels of pro-inflammatory cytokines such as IL-12 and monocyte chemoattractant protein 1 (MCP-1), despite chronic persistent hypercholesterolemia [65]. Although the relations refer to the animal model, similar pathophysiological relations characteristic of the atherosclerotic process were observed in humans with long-lasting DM [66]. Li et al. showed an increase in the expression of immune response factors such as NFκB, ICAM-1, IL6, and IL8 after treatment with TLR2 and TLR4 agonists in a group of patients with type 1 DM and advanced coronary atherosclerosis [67]. Enhanced secretion of these cytokines may accelerate the progression of the atherosclerotic plaque and increase the risk of its rapture. TLR receptors are responsible not only for signaling associated with infection but also for non-infectious factors, such as those resulting from excess visceral adipose tissue and the secretion of activating factors. Properties of these receptors may find application in the future as targets of treatment for macroangiopathic complications of DM. Therefore, it is important to adequately imitate its role in animal models [68]. 

#### 3.2.3. Pentraxins

Pentraxins (e.g., PTX3) constitute another group of factors that link the atherosclerotic process and the wound healing process. PTX3 is synthesized during neutrophil differentiation, it is accumulated in their mature follicles, and is ready for secretion under hypoxia in response to increased concentrations of proinflammatory cytokines such as TNF alpha, IL-1 beta, or the presence of lipopolysaccharides [69,70,71]. Therefore, PTX3 levels can be used not only as prognostic markers in myocardial ischemia but also in tissue recovery [72]. Increased levels of PTX3 were found in obese patients and in conditions of elevated insulin levels characteristic of insulin resistance, such as metabolic syndrome and DM [73,74]. The role of this molecule has not been well investigated in the context of wound healing. However, it seems that it can play an essential role in inflammation associated with tissue regeneration, and higher levels of it can be associated with delayed wound healing.

### 3.3. How Proteases Affect the Severity of the Atherosclerotic Process

#### 3.3.1. Matrix Metalloproteinases

Additional factors linking the pathogenesis of atherosclerotic lesions and the healing process are cathepsins and matrix metalloproteinases (MMPs), whose dominant role is the degradation of extracellular matrix components. Under physiological conditions, they are responsible not only for the reconstruction of vascular wall structures as well as formation and destabilization of the atherosclerotic plaque, but also for the regeneration of superficial injuries of the epidermis or mucosa [75,76,77]. Knock-out studies on animal models have provided knowledge about the role of these enzymes in the pathogenesis of atherosclerosis. Overactivity of different subtypes of metalloproteinases has been found in DM depending on the location of the pathological process. Increased levels of metalloproteinases -1,-2,-3 have been shown to be associated with markers of vessel wall stiffness in patients with type 1 DM and may represent a potential therapeutic target [78]. In contrast, in type 2 DM, elevated levels of MMPs-7 and -12 were associated with a more severe course of atherosclerosis and an increased frequency of coronary incidents [79]. Furthermore, atherosclerotic lesions in the arterial vessel wall showed increased activity of metalloproteinases MMP-7 in addition to MMP-1,-2,-3,-9 [80,81,82]. It seems that inhibition of these enzymes can reduce the risks of plaque rupture and further narrowing of the vessel. Moreover, overexpression of MMP-9 was associated with delayed wound healing, and MMP-1,-8,-9 were found in diabetic wounds [83,84,85,86]. During healing, there is a dynamic balance between the activity of metalloproteinases and tissue inhibitors of metalloproteinases (TIMPs) in the wound, especially in patients with DM. Yu Liu et al. demonstrated that in wound exudates of patients with DFS, the assessment of MMP-9 and TIMP-1 activity could help to evaluate patients with a high risk of delayed wound healing [87]. In a study evaluating carotid atherosclerotic plaque composition in patients with type 2 DM, overactivity of domain 17 (ADAM17) and MMP-9 was found to be associated with the inadequate expression of TIMP-3 via SirT1 [88]. 

#### 3.3.2. Cathepsins

Furthermore, cathepsins play an essential role in wound healing. Depending on their site of action, they affect not only cardiac remodeling (cathepsins B and S) but also bone (K) and adipose tissue (S) metabolism [89,90]. Moreover, cathepsin L plays a role in adipogenesis and insulin action. It was shown that inhibition of cathepsin L can result in the reduction of serum insulin, body weight gain, and protection of fibronectin from degradation [91]. An animal model should imitate the impaired function of ECM enzymes in DM. One of them is CatK −/− mice, which received bone marrow lacking cathepsin K. The mice presented with impaired functional recovery following hindlimb ischemia [92]. Cathepsins may also play a role in TLR9 receptor-mediated innate immunity, which may accelerate atherosclerotic lesions [93]. Diabetic patients have been shown to be deficient in the endogenous cathepsin inhibitor cystatin C, which suggests a correlation with increased serum cathepsin S. [94].

### 3.4. The Role of Procoagulant Factors in Atherosclerosis

The formation of atherosclerotic lesions would not be possible without the influence of procoagulant factors. Activation of the plasmin system appears to be particularly important in DM [95,96]. Plasminogen-activating factors have been found to mediate atherosclerotic plaque destabilization [97]. In the abovementioned mouse model of ApoE knockout, a lower plasminogen concentration was observed in addition to faster development of the atherosclerotic plaque. Factors inhibiting the NF-κB pathway may affect the inhibition of plasminogen activation. This can be observed in humans and experimental animals treated with drugs inhibiting this pathway, e.g., peroxisome proliferator-activated receptor gamma (PPAR-γ) agonists. An example of such an effect was demonstrated by Hao et al. on a mouse model with the use of rosiglitazone, which showed that angiotensin II inhibited plasminogen activating inhibitor 1 (PAI-1) and extracellular matrix (ECM) production, probably through interactions between PPAR-γ and TGF-β1/Smad2/3 and c-Jun N-terminal kinase (JNK) signaling pathways [98]. In clinical practice, two classes of drugs from this group are already used, i.e., fibrates (PPAR-α agonists), commonly used in diabetic dyslipidemia, and thiazolidinediones (PPAR-γ agonists), used in type 2 DM [98]. A rabbit model of hypercholesterolemia demonstrated an improvement in endothelial relaxation dependent on pioglitazone administration that was not directly related to lowering serum lipid levels [99]. 

### 3.5. Mast Cells and Their Significance in Plaque Formation

Mast cells play a special role among inflammatory cells involved in the development of atherosclerosis. They mature in the vessel wall under the influence of stem cell factor (SCF). They produce and secrete proteolytic substances which play a significant role in vessel wall remodeling. These proteolytic enzymes modify the composition and function of LDL and HDL lipoproteins [100]. In rodent mast cell cultures with a high content of LDL and HDL particles, accelerated formation of atherosclerotic plaques was observed on the inner vessel wall. On the other hand, after incubation of stimulated mast cells collected from the peritoneal cavity (peritoneal mast cells) with LDL particles, rapid proteolytic degradation of these particles was observed [101]. This degradation was caused by mast cell chymase present in cytosolic granules and was termed “granule-mediated uptake of LDL” [102]. It seems that the role of these cells depends on their phenotype which is also modified by hyperglycemia. Therefore, it is one of the mechanisms which ensures faster wound healing if the metabolic control of DM is accurate. 

Mast cells, which secrete paracrine and autocrine proinflammatory cytokines such as tumor necrosis factor (TNF), IL-33, and IL-6, participate in the mechanism of insulin resistance by activating kinases [103]. IL-37 (a member of the IL-1 7 family), continuously sustains chronic inflammation. It has been postulated that combining IL-37 (IL-1 family member 7) with the IL-18 receptor alpha (IL-18Rα) causes silencing of the innate and acquired immune response in type 2 DM, inhibiting the development of insulin resistance [104]. The impact of this modification on the wound healing process needs further investigation.

### 3.6. Lipid Disorders—A View beyond the Atherosclerotic Plaque 

The influence of lipid disorders on the development of the atherosclerotic process is indisputable in the present era of research. Foam cell formation on the macrophage core is mediated by endocytosis of oxidized lipoproteins via their receptors [105]. This process is multiplied by the glycation of apolipoproteins due to the defective action of insulin, impairing the ability of macrophages to cope with apoptosis [106]. Macrophages loaded with lipid masses secrete proinflammatory cytokines, chemokines, and reactive oxygen species, further stimulating inflammatory cells and causing the death of surrounding healthy tissues and impairing wound healing [105]. 

Two classes of receptors play an important role in maintaining readiness for foam cell activity, i.e., class A scavenger receptors (SR-A) and class B scavenger receptors (CD36). The role of these receptor types is to engulf modified lipids in the vessel wall for clearance. CD36 appears to be more important in activating monocytes and macrophages in the adipose tissue, liver, and arteries, leading to increased levels of soluble sCD36 in plasma and consequently maintaining the atherosclerotic process [107,108]. Increased levels of soluble forms of CD36 receptors have been found in the plasma of patients with DM and those with an unstable atherosclerotic plaque in carotid atherosclerosis. Several authors have demonstrated an association between sCD36 levels and glycemia measured as fasting glucose and HbA1C [109,110,111]. Therefore, the levels of these receptors may be considered markers of cardiovascular risk in the future.

Moreover, expression of the transmembrane lectin-like oxLDL receptor 1 (LOX-1), which is responsible for trapping oxidized LDL, has been found in endothelial cells, vessel wall smooth muscle cells, platelets, and macrophages [112]. The constant movement of foam cells between the bloodstream and the vessel wall is stimulated by a large influx of oxLDLs and overexpression of adhesion molecules on the endothelial surface due to their interaction with scavenger receptors on the foam cell surface. The macrophages loaded with oxLDLs have a reduced capacity to move to inflammatory sites and lymph nodes. The final result of this process is an increase in the number of foam cells. [113].

## 4. Animal Models versus Human Models—Similarities and Differences

### 4.1. Animal Model of Diabetic Wound—A Challenge

Wound progression and healing in DM in humans is a complicated process, as has previously been described in detail. As a result, the design of appropriate and potentially successful therapies requires optimal preclinical models of these processes. Several animal models have already been created, such as mouse, rat, hamster, and pig models (widely used models are given in Table 1). However, none of them perfectly reflects the process of diabetic wound healing in humans [114]. Most of them do not imitate all factors which are impaired in patients with DM (particularly immune reactions and metabolomic and transcriptomic changes specific to a certain organism). This should be considered during the analysis of the results.

In an established pig model (streptozotocin-injected Yorkshire pigs) of diabetic wound, the wounds healed within 18 days, which makes this model incoherent with the analogous process in humans [117]. Streptozotocin-induced models did not mimic type 1 DM because this disease is not caused by xenobiotic-mediated destruction of beta cells [118]. Similarly, type 2 DM in humans is not caused by the dysfunction of the leptin axis as in the case of ob/ob mice or db/db mice [119]. Although the stages of wound healing are similar and include coagulation, the inflammatory phase (immune cell infiltration, cytokine secretion), the proliferative phase (extracellular matrix generation, angiogenesis, epithelialization) and the remodeling phase (collagen crosslinking and reorganization), there are differences in skin ultrastructure and physiology between different species, which makes wound healing different in human and animals. In mice and rats, the skin is thin and fragile, whereas the subcutaneous layer consists of a special muscular striatum, panniculus carnosus (absent in humans), which causes more rapid contraction and facilitates wound healing [120]. Nevertheless, rodents are widely used in experimental models of diabetic wound healing and a number of them provided experience, which allows for further optimization of models in the aspects such as glycemic levels, the method of induction of DM, or duration of DM [121]. The advantages and disadvantages of different animal models used in the studies on diabetic wounds are given in Table 2.

### 4.2. How to Recreate the Neuropathic Component Observed in Patients with Long-Term Diabetes in an Animal Model?

A study conducted by the authors of this review showed that the duration of hyperglycemia in rats might reflect the long-term uncompensated course of DM in humans [123]. The results of pain threshold measurements confirm the onset of developing sensory neuropathy in the studied rats as early as day seven after administration of streptozotocin. In humans, the development of neuropathy is more spread out over time. In addition to the existence of DM per se, the development of complications is also influenced by other factors such as age, glycemic control, elevated cholesterol and triglyceride levels, hypertension, obesity, and smoking. EURODIAB IDDM, which was a study assessing the presence of DM complications in which more than three thousand patients from sixteen countries were treated with insulin, initially found neuropathy in 28% of patients, and an increase to 51.5% was recorded after a seven-year follow-up [124]. Therefore, the authors’ observations based on a rat model can provide answers to important clinical questions, because in an immeasurably shorter time it is possible to obtain the conditions for which researchers would need many years in the case of a human model. In many studies based on an animal model, Bujalska et al. used such a methodology to evaluate the process of developing diabetic neuropathy [125].

### 4.3. Ischemic Model—Is It Really a Chronic Wound Model?

Of note, the chronic wound model described in the vast majority of studies is in fact an acute wound model. One example is the rabbit model, in which two of the three arteries supplying the area with blood were ligated in the rabbit’s ear [126]. Next, a wound was created in the ischemic site. This model was mainly used to study the effect of growth factors on a wound. One modification of the rabbit model is the model proposed by Constantine and Bolton, describing the formation of an ischemic skin wound of a guinea pig [127]. This model involves the subcutaneous placement of a plunger tip of a disposable plastic syringe, wound closure, and ligation of the plunger base with a nylon fastening strap. In the experiments, a necrotic lesion developed after 9 h. Another model currently used in research for the analysis of chronic wounds is a model in which a rat or other rodent has a metal plate implanted into the skin. Then, with an external magnet, this plate compresses the skin, causing ischemia and ulceration. Over time, the blood supply is restored, which allows the inflow of inflammatory mediators [128]. These models do not fully reflect the wound in humans due to the physiological and anatomical differences between the species. They offer the possibility of controlled restoration of tissue perfusion. The above models allow for the creation of ischemic wounds, whereas in the case of chronic wounds in DM, there is an important neuropathic component, even if ischemic features coexist in the vascular bed. Moreover, these models do not imitate the activation of many impaired molecular pathways in patients with DM. This should be considered during the interpretation of the results and the analysis of potential utility for humans. 

Another concept for model selection is the anatomical recreation of the human skin model of a chronic wound in pigs [129]. As in the rabbit model, the blood supply to the wound site is also surgically restricted, causing tissue hypoxia. Due to the anatomical and physiological similarities to the conditions of wound healing in humans, this model offers very good conditions for testing. However, it is rarely used in preclinical studies due to the very high price of raising and keeping pigs. Choosing a rat as the basis for our model is also in line with the “replacement” principle recommended in animal model studies. However, skin transplantation onto animals may have an impact on wound healing due to the activation of inflammatory pathways and differences in MHC molecules.

### 4.4. Is It Possible to Translate the Conditions of Chronic Inflammation in DM from a Human to an Animal Model?

Most current animal models are based on the formation of acute wounds and then subjecting the wounds to factors responsible for the formation of chronic wounds, i.e., ischemia (e.g., damage associated with pressure) or DM. In the above study, a model imitating chronic wound conditions in patients with DM was presented [123]. The presence of DM means that different stages of wound healing exhibit changed characteristics. The inflammation phase is lengthened, the concentration of growth factors and number of cells in the proliferation phase are reduced, and neovascularization is impaired [130]. Due to the applied procedures, such as the maintenance of hyperglycemia for a long time, eventually causing sensory neuropathy, the use of bacterial lipopolysaccharide, and the inhibition of the participation of musculus panniculus carnosus in the species-specific process of rapid wound healing after an injury, it was possible to best reflect the conditions in the wound environment in a patient with long-term uncontrolled DM. If Lee et al. had not applied the above modifications, the healing time would have been much shorter (5–7 days) than that obtained in our study, and could not have accurately reflected the healing conditions of the chronic wound [131]. 

Another modification in out abovementioned study was the use of bacterial lipopolysaccharide placed directly on the wound as an additional factor for improving the model to recreate the conditions in the human model. The presence of DM in humans is a widely recognized factor for an increased risk of wound infection through hyperglycemia and also macro- and microangiopathy, causing worse tissue perfusion, as well as autonomic neuropathy, which is the cause of excessive drying of the skin and, as a consequence, its poorer regeneration. In their meta-analysis, Biancari and Giordano put forward a thesis about the existence of a positive correlation between the worse metabolic control of DM manifested in objective laboratory tests as a higher percentage of glycated hemoglobin and the frequency of postoperative complications in the form of wound infection after cardiac surgery [132]. For this reason, in patients with uncontrolled DM, multiple bacterial flora is reported, including multi-drug resistant strains such as Ps. aeruginosa. Therefore, the authors used this bacterium in the lipopolysaccharide rat model.

## 5. What Features Should the Best Model Have? Do We Know the Model Closest to the “Target”?

### 5.1. How to Prevent the Mechanism of Contraction Observed in Wounds Generated in the Musculus Panniculus Carnosus in Rats?

In the above paper, special modifications were applied to the model after the pilot study [133]. This included the use of silicone discs placed on the dorsal skin around the periphery of the wound to stabilize the wound healing process. As a result, a proliferation of neoplastic granulation tissue could be observed, which was not possible in the pilot study due to the different biomechanics of wound healing in rats. Galiano et al. [134] described such modifications in the literature. Another method for preventing premature wound healing by inhibiting constriction is that proposed by Yao et al. [135]. Their study was conducted in mice and featured a membrane used in, e.g., vacuum therapy, which was sutured to the shaved skin of the rodent’s dorsum, which prevented wound healing through skin contraction. According to Yao et al., after 48 h, the wound maintained 80% of the baseline area, while under normal conditions the wound decreased to 30% of the baseline area during this time, which roughly corresponds to the surface area reduction values obtained in our study. The undoubted advantage of the method is its low cost, as well as the simplicity of implementation. However, the problem is related to the durability of the membrane when it comes into contact with cage elements, other animals or even during research procedures.

Another concept for choosing a chronic model is the wound model presented by Reiss et al., who used a special Xanthan gum carrier applied to the wound. Recombinant collagenase (metalloproteinase-9) was suspended in Xanthan gum to a concentration parallel to that found in chronic wounds [136]. In the present study, the carrier delayed wound healing. The indisputable advantage of this scheme is the simplicity of using it, but due to the complex production process of the carrier and its significant costs, such a model could not be considered to be generally available or universal.

### 5.2. Does the Strain of Animal Matter in the Preparation of the Animal Model?

As mentioned before, the species of animals present different advantages and disadvantages in an animal model of diabetic wounds. The selection of the appropriate strain is also important. For instance, Zucker Diabetic Fatty (ZDF) rats or Zucker-Diabetic-Sprague Dawley rats are more specific for investigation of type 2 DM than Wistar-Kyoto (WKY) rats (used in studies on hypertension) [137,138,139]. However, modified WKY rats have also been used in diabetic studies [140]. Importantly, the arrangement of the experiment requires careful strain selection. 

## 6. Conclusions

The risk of a wound in a diabetic patient lies not only in the fight against potential infection but above all in its long-term persistence, which, with a decreased immune barrier, exposes the patient to life-threatening complications such as sepsis. Patients with DM are also at greater risk of wound tissue resection due to severe infection or irreversible ischemia of the wound area. Since consequences such as high amputation significantly shorten the life of patients with DM, intensive treatment is needed as early as possible in the course of the disease to prevent its chronicity [141]. Hyperglycemia impairs many of the described pathways, which can cause a delay in wound healing. Intensive research is conducted worldwide and its aim is to develop medicinal products which would result in the closure of the wound as quickly as possible and would also prevent the development of infection. Preparation of such molecules requires the development of an animal wound model that reflects as closely as possible the conditions and molecular differences of a chronic wound in a patient with DM. In individual subsections, we described the molecular mechanism of the development of endotheliopathy, immunopathy, and neuropathy. Additionally, we presented the difficulties researchers may encounter when choosing an animal species for the model, the way of creating the wound, and even the mapping of the desired dominant etiopathological factor of the wound. We also discussed what innervations may be applied to obtain the best imitation of chronic wound in an animal model. Such information can help in designing an adequate animal model which could ensure the development of new, safe, and effective medicinal products for the treatment of chronic wounds in patients with DM.

## Figures and Tables

**Table 1:** Selected animal models of diabetic wound healing (based on [114]). NOD—non-obese diabetic; BB—bio-breeding; 1—streptozotocin-mediated destruction of beta-islets, generating type 1 diabetes; 2—a spontaneous development of autoimmune reactions leading to type 1 diabetes; 3—selected line of hamsters presenting with glucosuria and exhaustion of beta cells [115]; 4—leptin receptor deficiency, severe obesity; 5—a point mutation in the leptin receptor gene, severe obesity; 6—recombinant strain, polygenic background of diabetes, moderate obesity; 7—fa gene homozygous mutation in the leptin receptor gene OB-R, resistance to leptin [116].

	Mice	Rats	Other Animals
**Type 1 diabetes**	Streptozotocin-induced ^1^ diabetic mice NOD mice ^2^	Streptozotocin-induced ^1^ diabetic rats BB rats ^2^	Chinese hamster ^3^
**Type 2 diabetes**	Obese *ob/ob* mice ^4^ *db/db* mice ^5^ NONcNZO mice ^6^	Zucker *fa/fa* rats ^7^	

**Table 2:** Advantages and disadvantages of different animal models in studies on human diabetic wounds (based on [114,122]).

Model	Advantages	Disadvantages
**Mice**	Relatively low cost High rate of breeding Short time of a single experiment Easy transgenic model generation	Differences in: mechanism of wound healing (**contraction**) skin ultrastructure innate and adaptive immune systems
**Rat**	Similar to mice Larger dimensions, better endurance—more stable experimental conditions	Different mechanism of wound healing (**contraction**) A paucity of specific reagents (compared to mice)
**Rabbit**	Relatively low costs Rapid breathing Similar response to different factors as in human skin Ear model (contralateral ear as a control)	Difficulties in transgenic organisms generation A paucity of specific reagents
**Pig**	Anatomical and physiological similarities with humans Similar mechanism of wound healing as in humans—**re-epithelialization and granulation**	Incoherence with humans (rapid wound healing) High cost Long time of a single experiment

## Data Availability

Not applicable.
